# Simulation of melting paraffin with graphene nanoparticles within a solar thermal energy storage system

**DOI:** 10.1038/s41598-023-35361-8

**Published:** 2023-05-26

**Authors:** M. Jafaryar, M. Sheikholeslami

**Affiliations:** 1 Renewable Energy Systems and Nanofluid Applications in Heat Transfer Laboratory, Babol Noshirvani University of Technology, Babol, Iran; 2 Department of Mechanical Engineering, Babol Noshirvani University of Technology, Babol, Iran

**Keywords:** Nanoscience and technology, Nanoscale materials

## Abstract

In this paper, applying new structure and loading Graphene nanoparticles have been considered as promising techniques for enhancing thermal storage systems. The layers within the paraffin zone were made from aluminum and the melting temperature of paraffin is 319.55 K. The paraffin zone located in the middle section of the triplex tube and uniform hot temperatures (335 K) for both walls of annulus have been applied. Three geometries for the container were applied with changing the angle of fins (α = 7.5°, 15° and 30°). The uniform concentration of additives was assumed involving a homogeneous model for predicting properties. Results indicate that loading Graphene nanoparticles causes time of melting to decrease about 4.98% when α = 7.5° and the impact of ϕ improves about 5.2% with reduce of angle from 30° to 7.5°. In addition, as angle declines, the period of melting decreases around 76.47% which is associated with augmentation of driving force (conduction) in geometry with lower α.

## Introduction

Renewable energy sources, natural ventilation, and heating and cooling systems have all been taken into account from the standpoint of sustainable development. As a result, research has focused on a number of subjects, including the study of free convection. In this context, a number of researchers have investigated a variety of strategies to reduce energy usage in a variety of devices. These solutions have included the use of nanofluids, fins, and other techniques. Additionally, one of the most important issues with eco-friendly architecture and residential spaces is how to keep people comfortable year-round by heating and cooling these areas during the cold and hot seasons. Therefore, the greatest possible savings can be made when special considerations are made for things like window shape, size, type of construction material, climate, etc. By collecting, storing, and transferring solar energy as heat removal in the summer, passive building design makes use of walls, windows and floors. The use of fossil fuels and the need for heating and cooling systems are decreased by the inert design. Use of a Trombe wall (solar wall) is one technique for passive heating. Two arrangements of fractal fins with various levels of bifurcation were taken into consideration by Luo^1^. They discussed the Phase change material (PCM)'s melt heat-transfer process is affected by fin spacing. According to the findings, using complex fractal fins instead of the traditional fractal fin resulted in a 68% shorter time for PCM to completely melt in the heat exchanger^1^. Cheng et al.^2^ utilized a numerical approach to model the phase transition, a composite PCM made of copper oxide and paraffin was employed. According to their study, when the length ratios of the 3 sets of fins were 0.3333, 0.6 and 1, the melting times were decreased by 33.06%, 22.55% and 16.7% and the solidification times were decreased by 47.33%, 50.99% and 57.54% when compared to the design without fins. Fins with considerable length disparities that are unequal in length are chosen when only one melting process is taken into account. Equal-length fins are preferable when seeing the entire development of storing and releasing heat. PCM charging in a three-pipe was researched by Natteri^3^, and the findings showed that more fins in the bottom area was the ideal arrangement. The easiest technique to raise thermal efficiency is to add fins. However, there are definite drawbacks to these techniques. The weight of the system is increased by the fins, and the amounts energy is decreased as the percentage of PCM is decreased.

Abdullah et al.^4^ utilized the paraffin for improving the solar still unit. Their results showed that employing electrical heaters and PCM with nanoparticles simultaneously increased daily water productivity by 196%. Mahdi et al.^5^ scrutinized the treatment of heat nano-enhanced PCM within the charging in a triplex-tube duct made of porous foam. Relying on the volumetric nanoparticle concentration and the structure of the foam they discovered that adding nanoparticles to metal foams can speed up melting by as much as 90%. Notably, adopting uneven fins to increase the heat transfer impact can be employed to overcome the issue of the uneven solid–liquid interface generated. Chen et al.^6^ used RT42 as the PCM to investigate the effects of double fins with uneven lengths on heat transmission and melting rate. In the square cavity, they suggested mounting two fins of different lengths. The outcomes showed that the phase interface's inhomogeneity can be improved by using long fins to encourage convection. In order to simulate the brick’s latent heat storage, Dabiri et al.^7^ employed a container in the center and 5 circular air holes. It was studied how much sensible and latent heat PCM could hold onto during Tehran's warmest and coldest days of 2016. In contrast to summer, they found that winter is a better time to use latent heat storage. The interior temperature variations of different bricks, counting one with air holes, and a straightforward brick, were also compared by the researchers. Some researchers have utilized this technique because experimental approaches are accurate. To ensure the least amount of PCM volume loss and the largest amount of natural convection contribution, nanoparticle concentration and porosity should also be carefully tuned. The same authors achieved comparable outcomes for the solidification example^8^. The findings showed that in the presence of metal foam scattering nanoparticles could reduce time by up to 96%. Due to benefits including simplicity of manufacturing, low price, and huge heat exchange area, researchers have analytically and numerically studied the addition of a metal fin. When heat is stored, the existence of fins encourages convective heat release. Numerous experiments have supported this, including Tian^9^, where copper, aluminum and steel fins were placed inside the container, and the square cavity's PCM melting durations were shortened by 41.6%, 40.1%, 41.0%, and 37.2%. Qiu et al.^10^ developed a bionic bone hierarchical porous material and integrated it with a novel PCM. They conducted experiments to measure the thermal contact resistance between the skeleton and composite and determined that the composite had a melting point of 54.75 °C. Nazanin Variji et al.^11^ did numerical research to determine how PCMs have foam-metal inserts affect heat transmission. Various inclination angles and porosities were contrasted, and the outcomes showed that raising the inclination angle helped the PCM's heat transmission and natural convection, and compared to PV-PCM, the electrical efficiency and average temperature rose by 9.8% and 6.8% respectively. Qiu et al.^12^ have investigated the significance of using nano/micro PCMs in solar thermal systems. Their study has shown that incorporating fins and increasing the porosity of the domain through foam can improve the thermal improvement degree and discharge rate.

Regarding literature, the attempts for improving the geometry of heat storage units have been made by several researchers and this topic has become popular among them because of the importance of energy saving. In this paper, the efficacy of applying complex tank for the paraffin zone has been studied in the existence of the triplex tube. Graphene nanoparticles have been mixed with paraffin to boost the performance. Three levels for angle of fins have been applied to generate various geometries for the container and simulations were done for both PCM and NEPCM. The concentration of additives is 0.03 and homogenous mixture has been assumed in deriving the features of NEPCM. The governing equations and assumptions were mentioned in section “New complex shape for energy storage involving mixture of paraffin and Graphene nanoparticles”. The configurations of the grid and validation test were studied in the first part of section “Results and discussion”. Moreover, the influences of loading nanoparticles and changing geometry have been scrutinized in this section. The fourth section belongs to the conclusion of present work.

## New complex shape for energy storage involving mixture of paraffin and graphene nanoparticles

**Figure 1:**
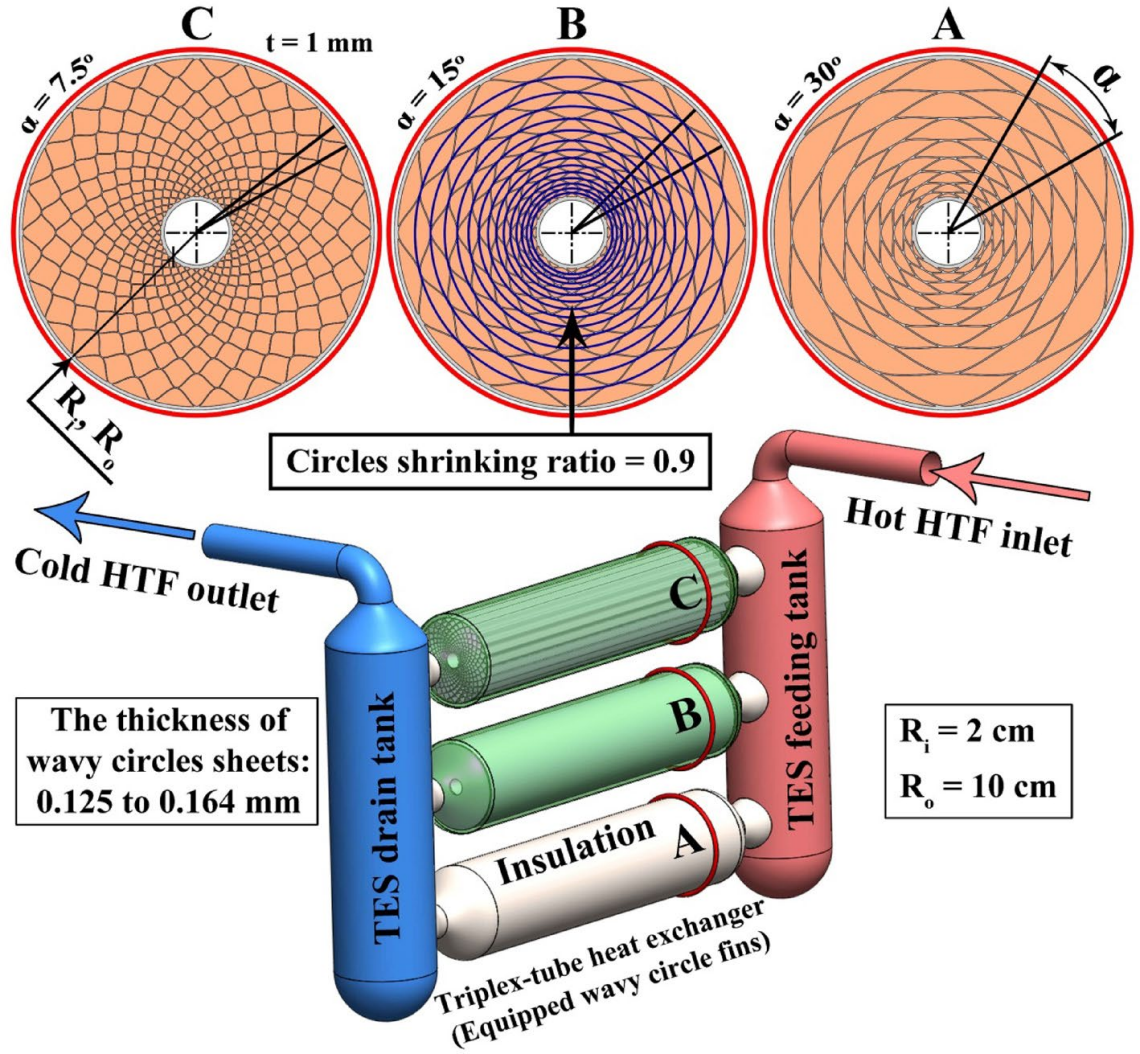
Thermal storage system with triplex tube involving innovative structure of paraffin zone.

In present work, triplex tube with radius of R_i_ = 2 cm and R_o_ = 10 cm has been utilized (see Fig. 1). The annulus region was filled with paraffin and melting temperature is 319.55 K. As shown in Fig. 1, the wavy radial fins were applied with various angles (α = 7.5°, 15° and 30°). The temperatures of the inner and outer walls of the annulus region are 335 K and initial temperature of system is 315 K. The structure was made from aluminum and to enhance the thermal performance, Graphene nanoparticles were dispersed into paraffin with a fraction of 0.03. The features of applied material and formulations for deriving the properties of NEPCM have been summarized in Table 1^13–15^. Unsteady laminar flow within 2D domain has been assumed and below equations should be considered^16,17^:

1 $$ k_{NEPCM} \nabla^{2} T - \frac{{\partial \left( {\chi \rho L} \right)_{NEPCM} }}{\partial t} = \left[ {\frac{\partial T}{{\partial t}} + \mathop V\limits^{ \to } \, \cdot \,\nabla T} \right]\left( {\rho C_{p} } \right)_{NEPCM} $$

2 $$ \chi = \left\{ \begin{gathered} \frac{{T_{s} - T}}{{T_{s} - T_{l} }}\,\,\,\,\,\,\,\,\,\,\,\,\,\,\,\,T_{s} \, < T < T_{l} \hfill \\ \hfill \\ 0\,\,\,\,\,\,\,\,\,\,\,\,\,\,\,\,\,\,\,\,\,\,\,\,\,\,\,\,\,\,\,\,\,\,\,\,\,\,T < T_{s} \hfill \\ \hfill \\ 1\,\,\,\,\,\,\,\,\,\,\,\,\,\,\,\,\,\,\,\,\,\,\,\,\,\,\,\,\,\,\,\,\,\,\,\,\,\,T > T_{l} \hfill \\ \end{gathered} \right. $$

3 $$ \left[ {\frac{\partial v}{{\partial t}} + \mathop V\limits^{ \to } \, \cdot \,\nabla v} \right]\rho_{NEPCM} = \left( {\beta \rho } \right)_{NEPCM} \left( {T - T_{ref} } \right)g + \left( {\left( {\nabla^{2} v} \right)\mu_{NEPCM} - \nabla P} \right) + \,\frac{{\left( {1 - \chi } \right)^{2} }}{{\varepsilon + \chi^{3} }}vC\,, $$

4 $$ \left[ {\mathop V\limits^{ \to } \, \cdot \,\nabla u + \frac{\partial u}{{\partial t}}} \right]\rho_{NEPCM} = \left( {\left( {\nabla^{2} u} \right)\mu_{NEPCM} - \nabla P} \right) + \,\,\frac{{\left( {1 - \chi } \right)^{2} }}{{\varepsilon + \chi^{3} }}Cu,\varepsilon = 10^{ - 3} ,C = 10^{5} $$

5 $$ \nabla \, \cdot \,\mathop V\limits^{ \to } = 0 $$

**Table 1:** Properties of material and formulation of NEPCM^13–15^.

Property	PCM^13^	Graphene^14^	NEPCM	Aluminum^15^
ρ (Kg/m^3^)	857	2200	${\rho }_{NEPCM}=\phi {\rho }_{P}+\left(1-\phi \right){\rho }_{PCM}$	2710
Cp (J/(KgK))	2200	790.1	${\left(\rho {C}_{P}\right)}_{NEPCM}=\phi {\left(\rho {C}_{P}\right)}_{P}+\left(1-\phi \right){\left(\rho {C}_{P}\right)}_{PCM}$	1256
k (W/(mK))	0.157	5000	${k}_{NEPCM}={k}_{PCM}\left[\frac{{k}_{P}-2\phi \left({k}_{PCM}-{k}_{P}\right)+2{k}_{PCM}}{{k}_{P}+\phi \left({k}_{PCM}-{k}_{P}\right)+2{k}_{PCM}}\right]$	180
β (1/K)	0.0006	0.07	${\left(\rho \beta \right)}_{NEPCM}=\left(1-\phi \right){\left(\rho \beta \right)}_{PCM}+\phi {\left(\rho \beta \right)}_{P}$	N/A
μ (Kg/(ms))	0.0067	N/A	${\mu }_{NEPCM}=\frac{{\mu }_{PCM}}{{\left(1-\phi \right)}^{2.5}}$	N/A
T_s_ (°C)	44.5	N/A	44.5	N/A
T_l_ (°C)	48.3	N/A	48.3	N/A
L (KJ/Kg)	178.78	N/A	${\left(L\rho \right)}_{NEPCM}=\left(1-\phi \right){\left(L\rho \right)}_{PCM}$	N/A

**Figure 2:**
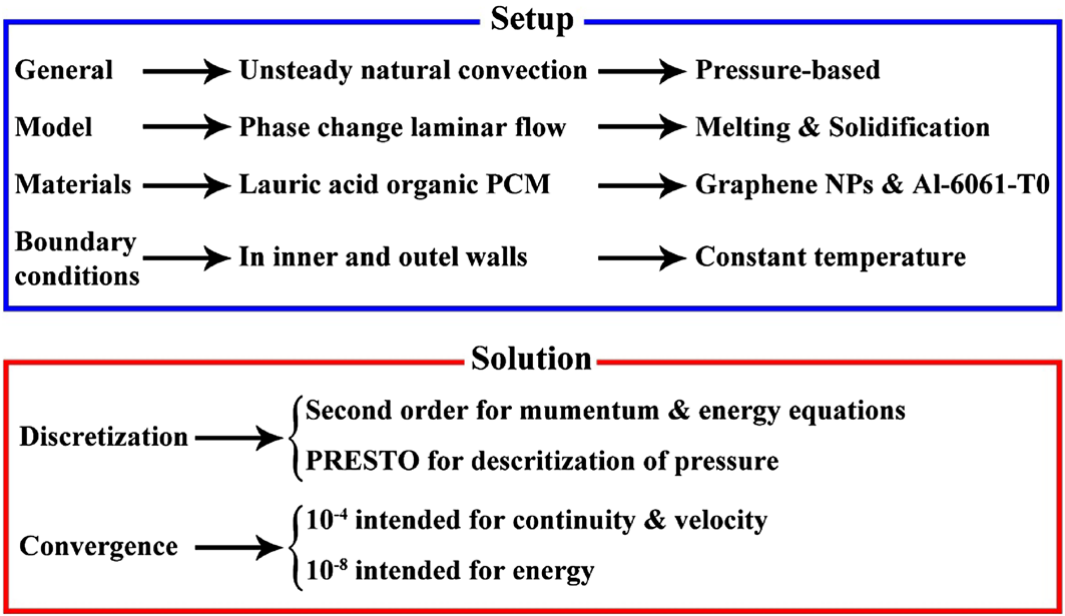
The selected techniques in modeling.

In above equations, $\chi$ is liquid fraction (LF) of paraffin. Also, the features of NEPCM have been calculated according to the formulation mentioned in Table 1. For modeling above equation within 2D geometry which was depicted in Fig. 1, finite volume method was hired utilizing ANSYS FLUENT. Figure 2 demonstrates the applied techniques in modeling via software. For discretization of pressure equations, PRESTO algorithm was applied. The values of residual were less than 10^–4^ for continuity and less than 10^–8^ for temperature. Boundary conditions for inner and outer walls are constant temperature. In selecting the model, phase change laminar flow was applied. In materials section, properties of NEPCM based on homogeneous models have been reported.

## Results and discussion

It seems to scientists that performance of thermal storage in charging steps rely on the structure of the PCM zone and with the installation of an extended surface, higher productivity of the system can be achieved. This is true that adding nanoparticles in such pure paraffin with low conductivity is helpful to expedite the process. In this article, a triplex tube structure has been utilized in which the middle section is filled with paraffin. The paraffin region was equipped with wavy circular fins. There exists one geometrical factor (angle of fins) as variable in this simulation and three levels were considered for α (7.5°, 15° and 30°). The temperature of the domain at the start of the process is 315 K and the temperature of melting for utilized paraffin is 319.55 K. Both walls of the annulus region have the same temperature of 335 K during the process. The low amount of conductivity of pure paraffin can cause huge problems, so, Graphene nanoparticles were loaded into base PCM to remove this problem. The offered structure can be utilized for saving solar energy during the day and finally the reverse process can provide warmer water during the night. Fins were made from alumina in this system. The concentration of Graphene nanoparticles have been considered uniformly which means that slip velocity has been neglected in modeling. The impact of buoyancy force during melting has been involved and two dimensional systems have been assumed.

### Verification of numerical approach

**Figure 3:**
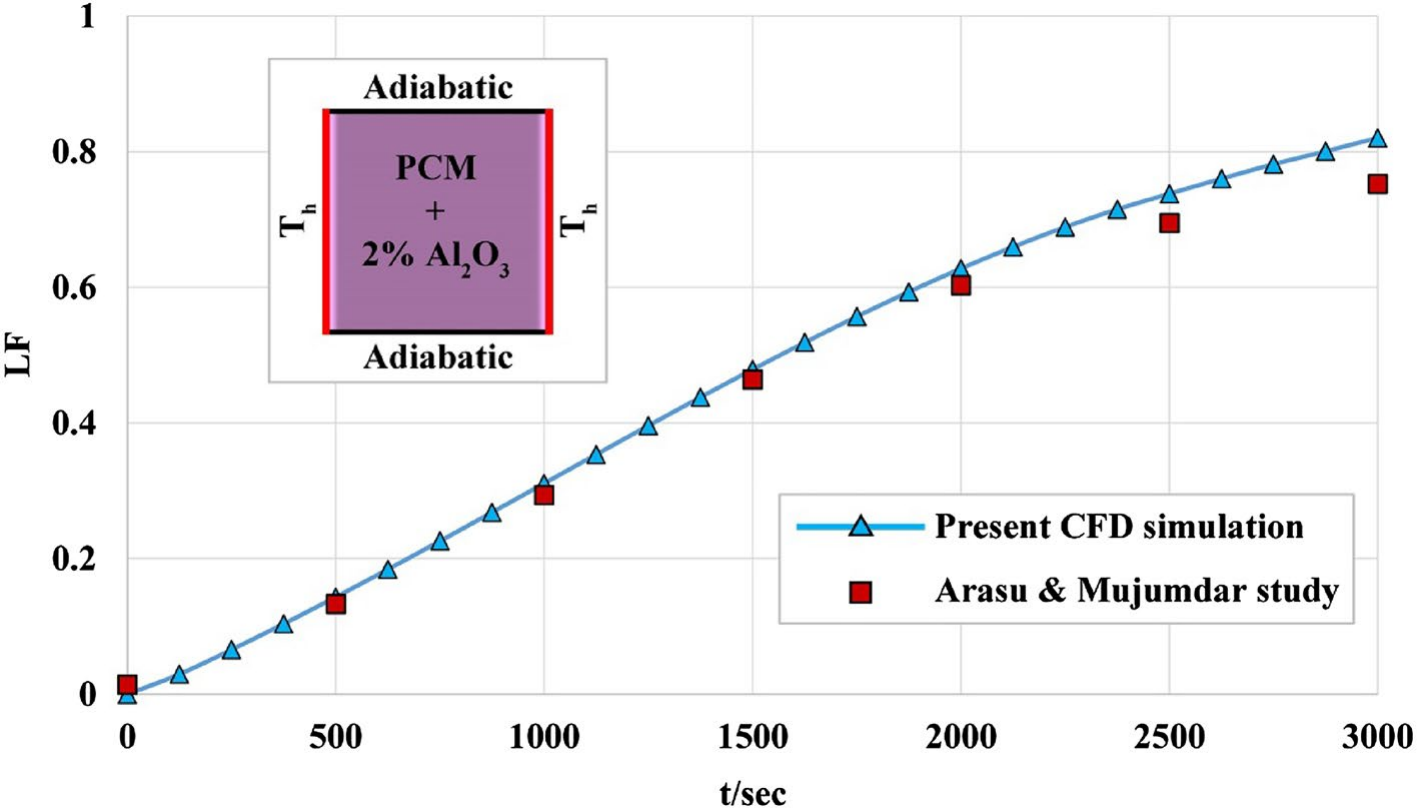
Validation test with data of previous work^18^.

For testing the accuracy of utilized assumptions in modeling, previous work of Arasu and Mujumdar^18^ were simulated. They considered melting of PCM two dimensional domains in the existence of alumina nanoparticles. There exist two vertical hot walls and horizontal walls that were insulated. The amounts of LF have been compared in Fig. 3 and outputs show that good agreement exists between the present data and previous outputs. Therefore, the utilized numerical method has reasonable accuracy and can be utilized for present study.

### Mesh generation and its details

**Figure 4:**
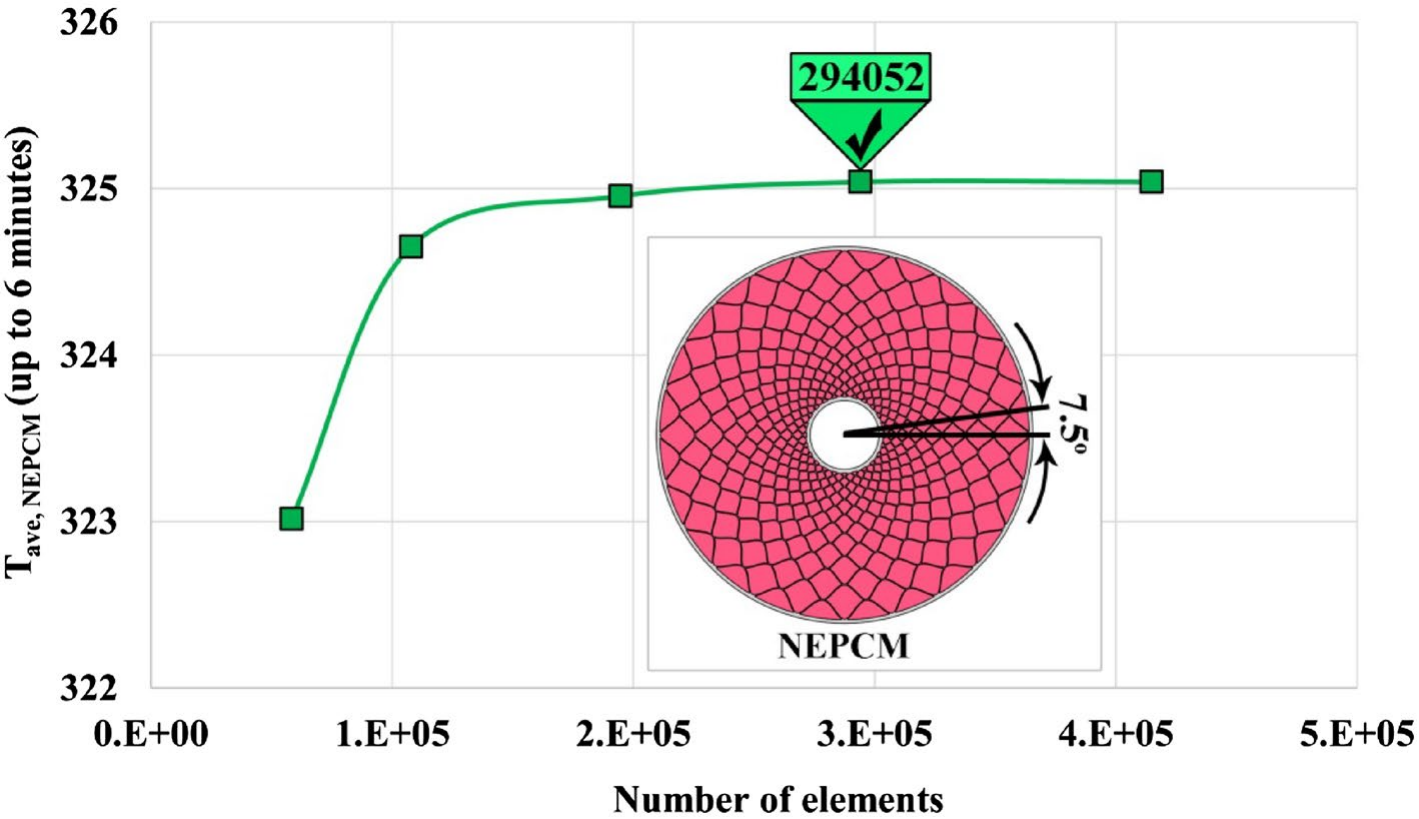
Obtaining the best number of element for α = 7.5°.

**Figure 5:**
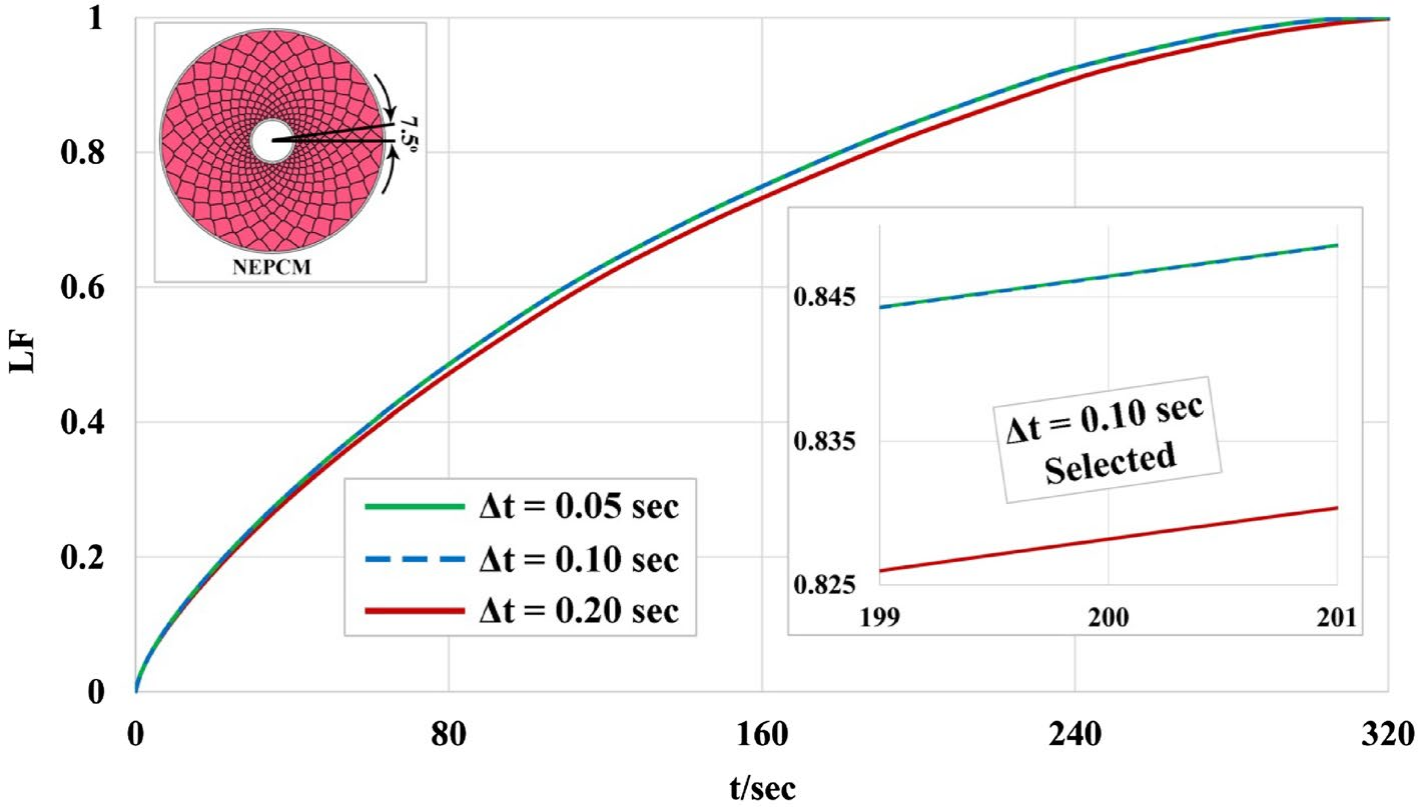
Obtaining the best amount of time step for α = 7.5°.

**Figure 6:**
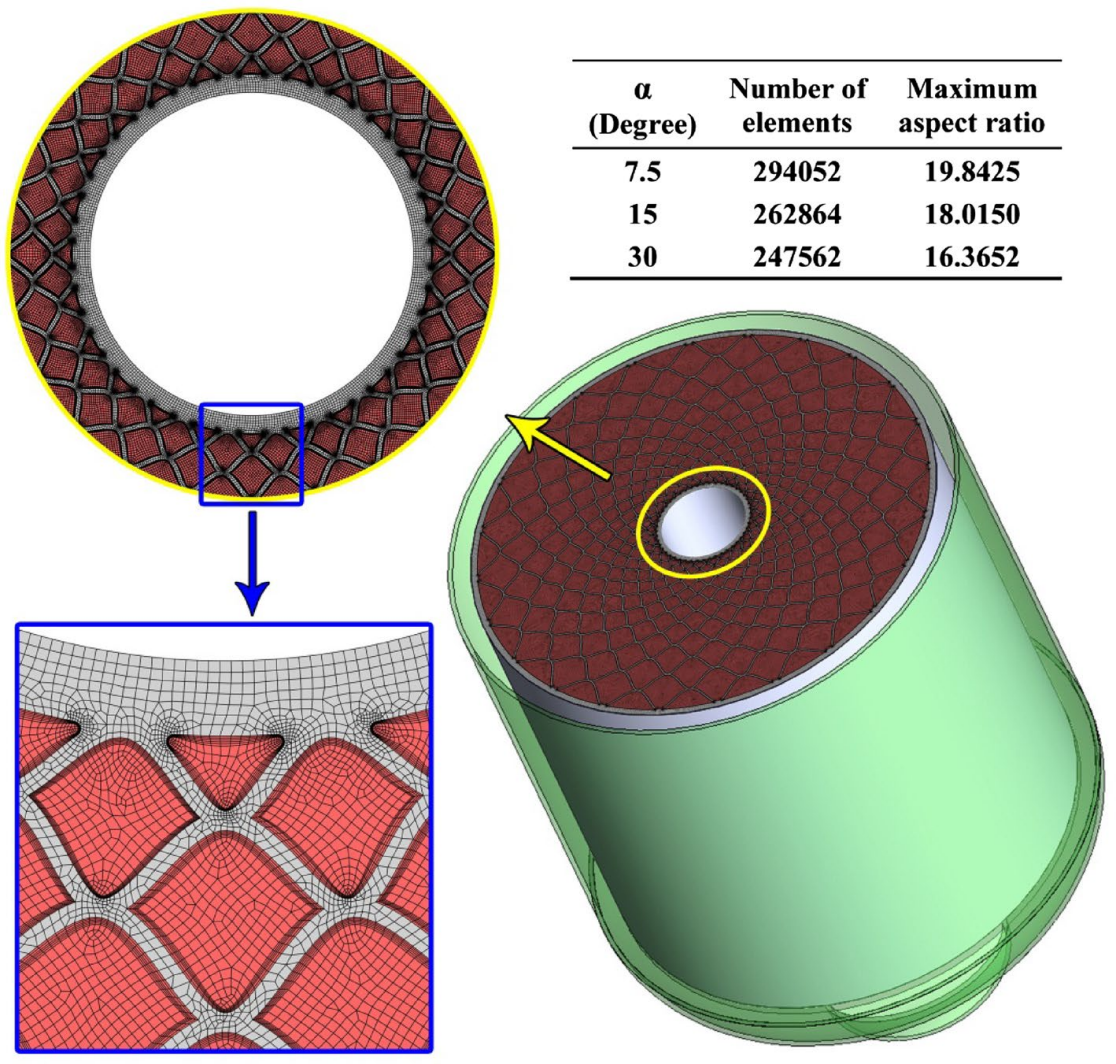
Details of utilized grid and its configuration.

Because of the unsteady inherentity of the melting process, the grid generation and selecting a suitable amount for time step plays a key role in reducing computing price. The structure grid has been utilized and various numbers of elements have been applied as reported in Fig. 4. Five arrangements of the grid have been scrutinized and average values of T_NEPCM_ in time of 6 min were reported. The outputs illustrate that choosing 294,052 elements results in an optimized grid because utilizing more elements has no sensible change in outputs. Also, three levels of time step have been tested and variation of LF was demonstrated in Fig. 5. According to this figure, selecting Δt = 0.1 s leads to the best value of this factor which provides the lowest computing price. To illustrate the configuration of grid, the grid of one case was demonstrated in Fig. 6. The number of elements for various cases are 294,052, 262,864 and 247,562 when α = 7.5°, 15° and 30°, respectively. The maximum aspect ratios for these ranges of angles were 19.8425, 18.015 and 16.3652.

### Influence of α on melting process

**Figure 7:**
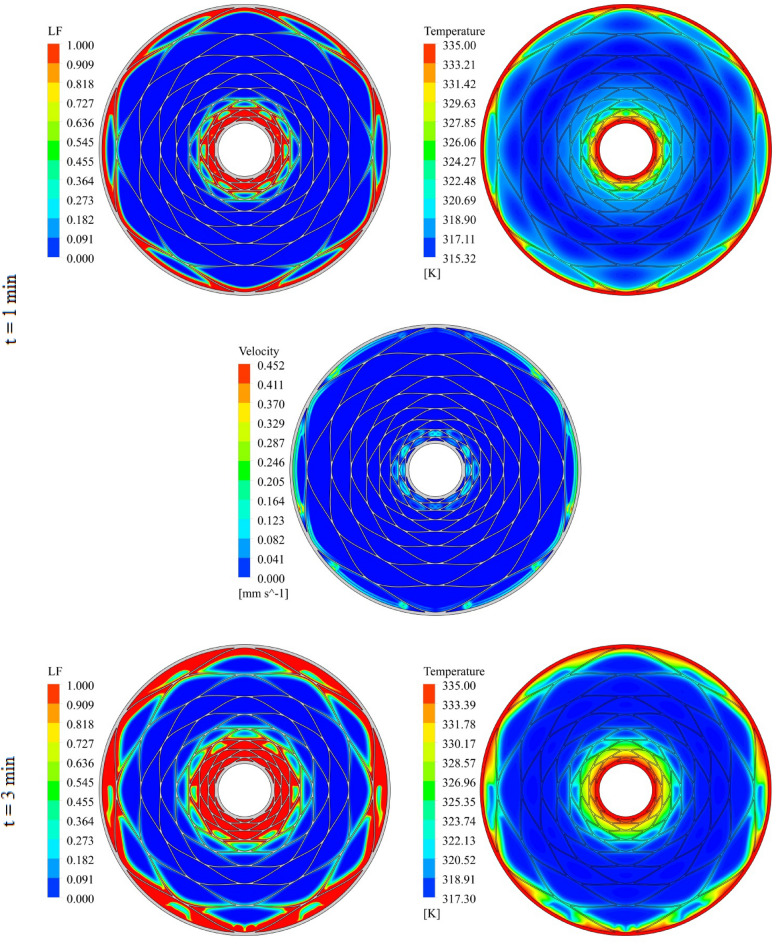

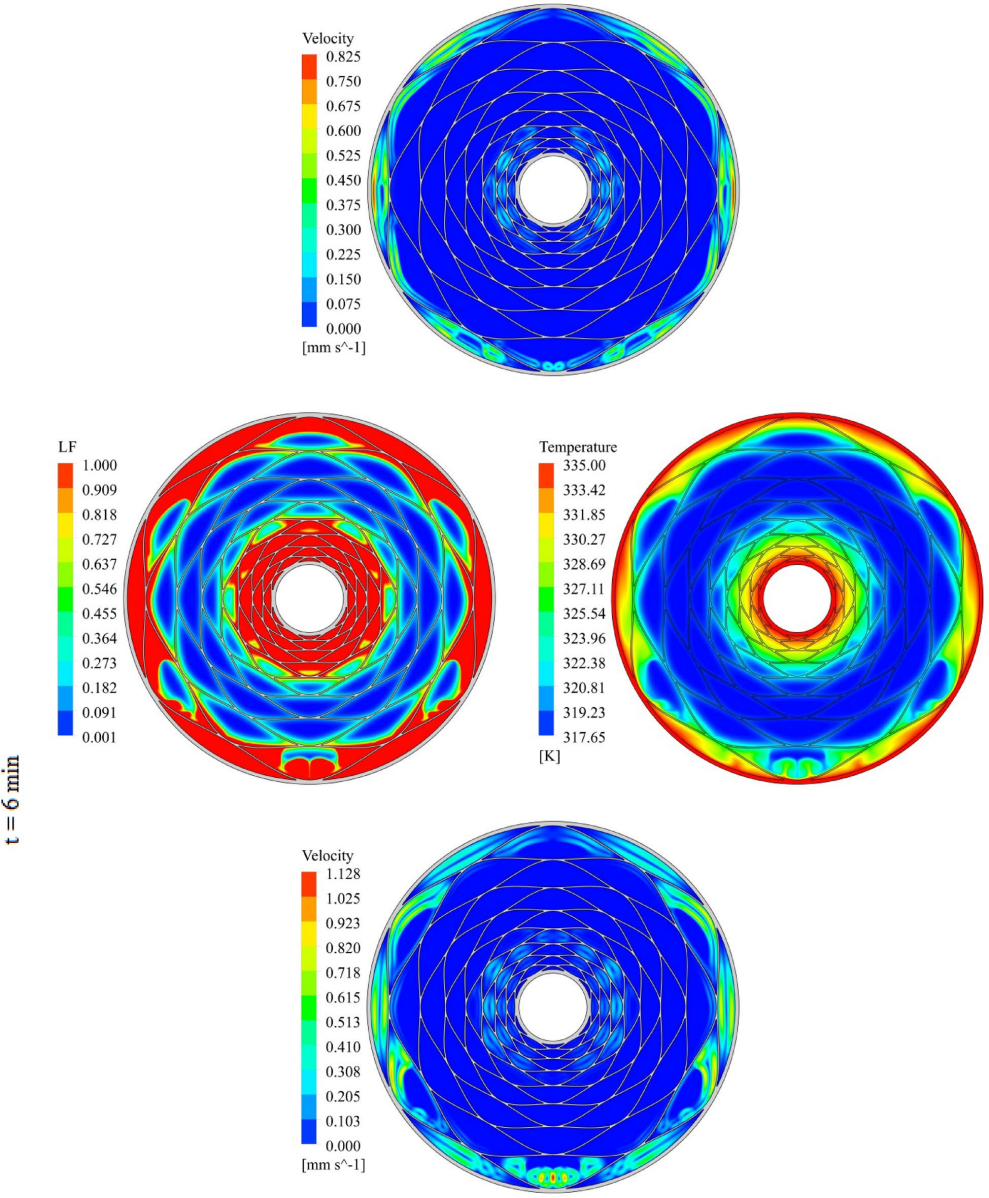
Progress of melting in presence of new structure when $\phi = 0.03,\,\;\alpha = 30^\circ$.

**Figure 8:**
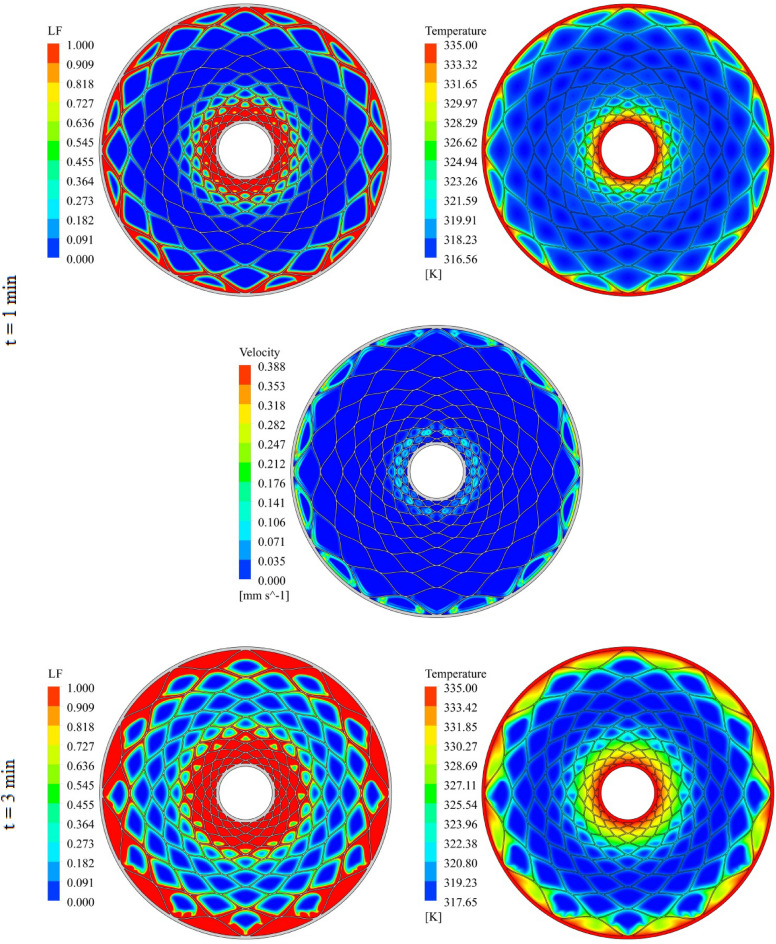

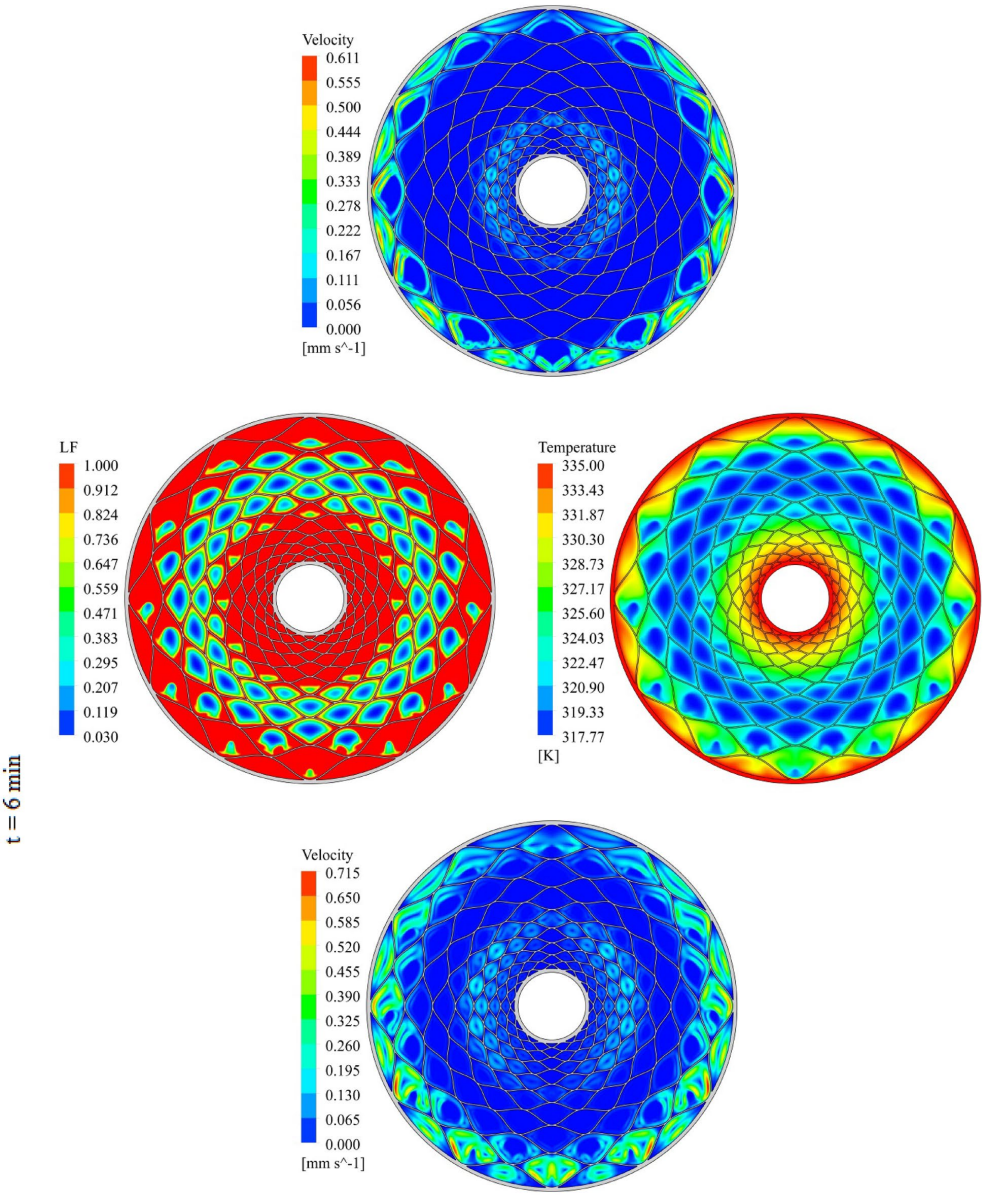
Progress of melting in presence of new structure when $\phi = 0.03,\,\;\alpha = 15^\circ$.

**Figure 9:**
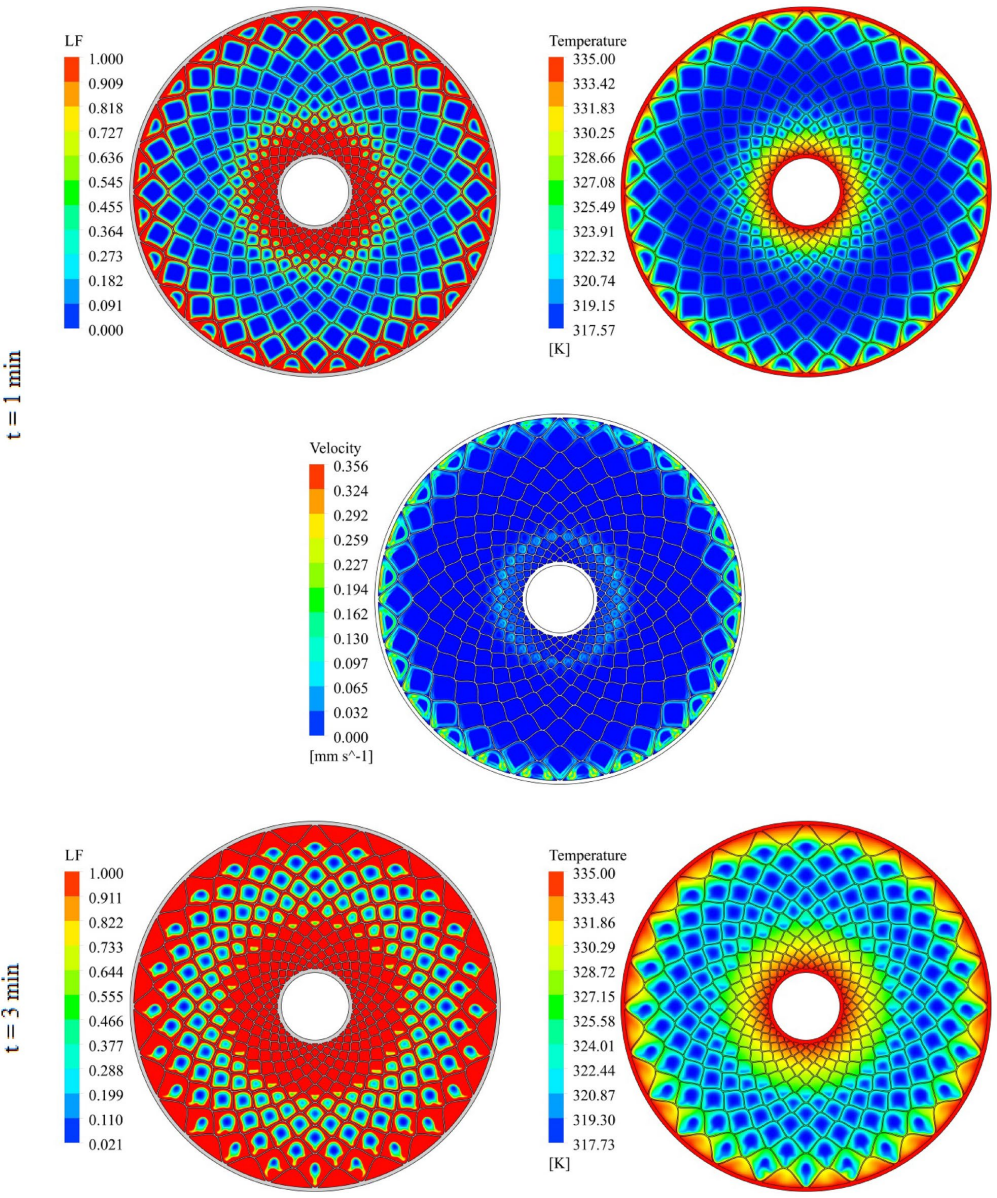

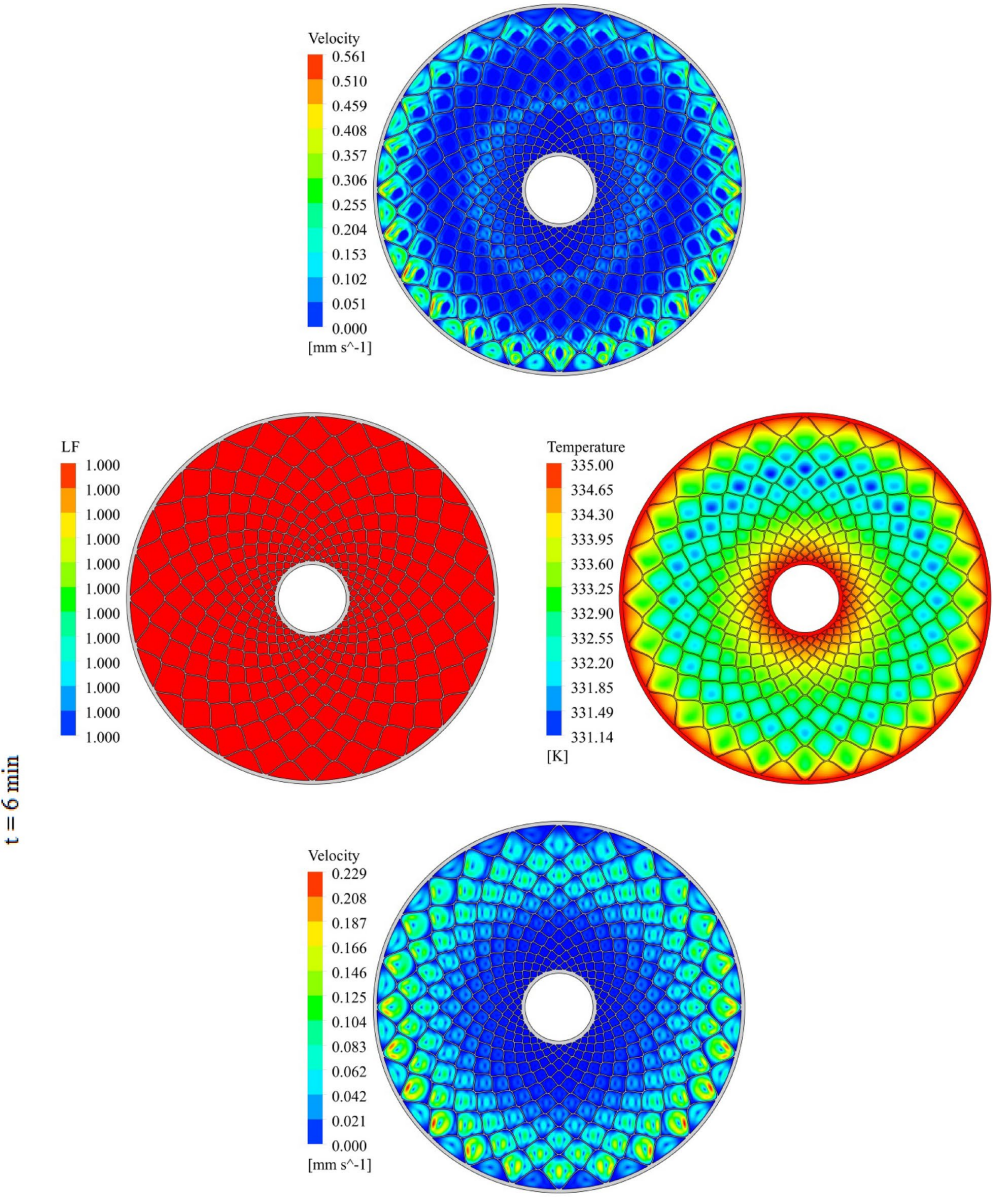
Progress of melting in presence of new structure when $\phi = 0.03,\,\;\alpha = 7.5^\circ$.

The geometry of container can change the treatment of NEPCM and this section belongs to show the result of organization of fins on charging. The distributions of velocity, T and LF for three time level during melting have been demonstrated in Figs. 7, 8 and 9. The temperature of walls is greater than other places, so, melting starts from these regions. The contours for three levels of time (1 min, 3 min and 6 min) have been reported. Adding nano-powders can enhance the LF about 154.17%, 158.43% and 10,916% when t = 1 min, 3 min and 6 min, respectively. Also, the temperature enhances about 0.81%, 1.38% and 3.54%, respectively. As angle decreases the amount of LF increases around 144.63%, 149.68% and 102.88% when t = 1 min, 3 min and 6 min, respectively. As time increases, LF and T grow about 151.37% and 3.95% when minimum level of angle has been utilized. With changing angle from 30° to 15° and 7.5°, the required time declines from 1377 to 660 s and 324 s. The quickest process occurs for the minimum level of α. Due to presence of denser fins near the inner tube, the melting around that region is faster than the region near the outer wall. The magnetite of velocity near the region with greater temperature gradient is greater.

### Variation of average LF, T and required heat for melting

**Figure 10:**
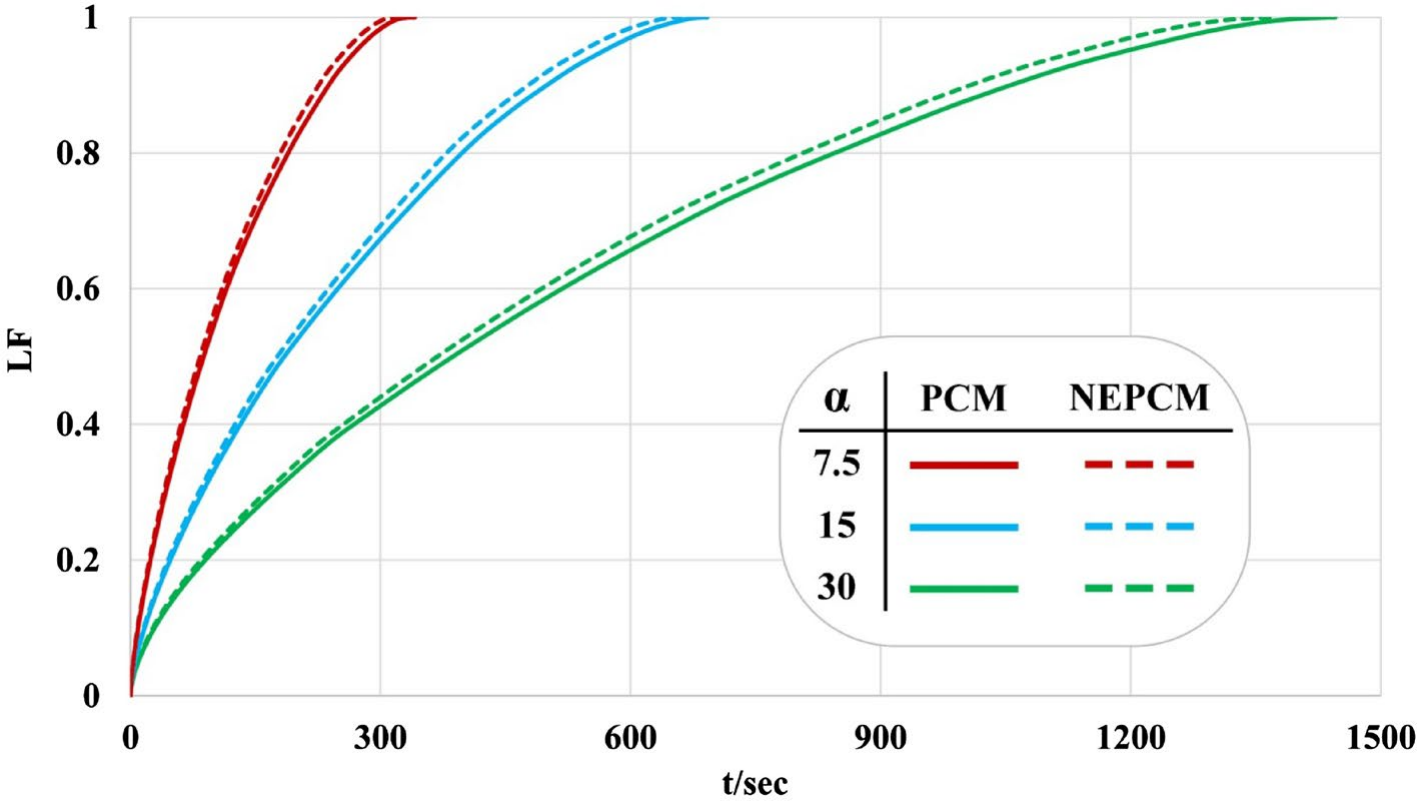
Effect of dispersing nanoparticle for the various structures in view of liquid fraction.

**Figure 11:**
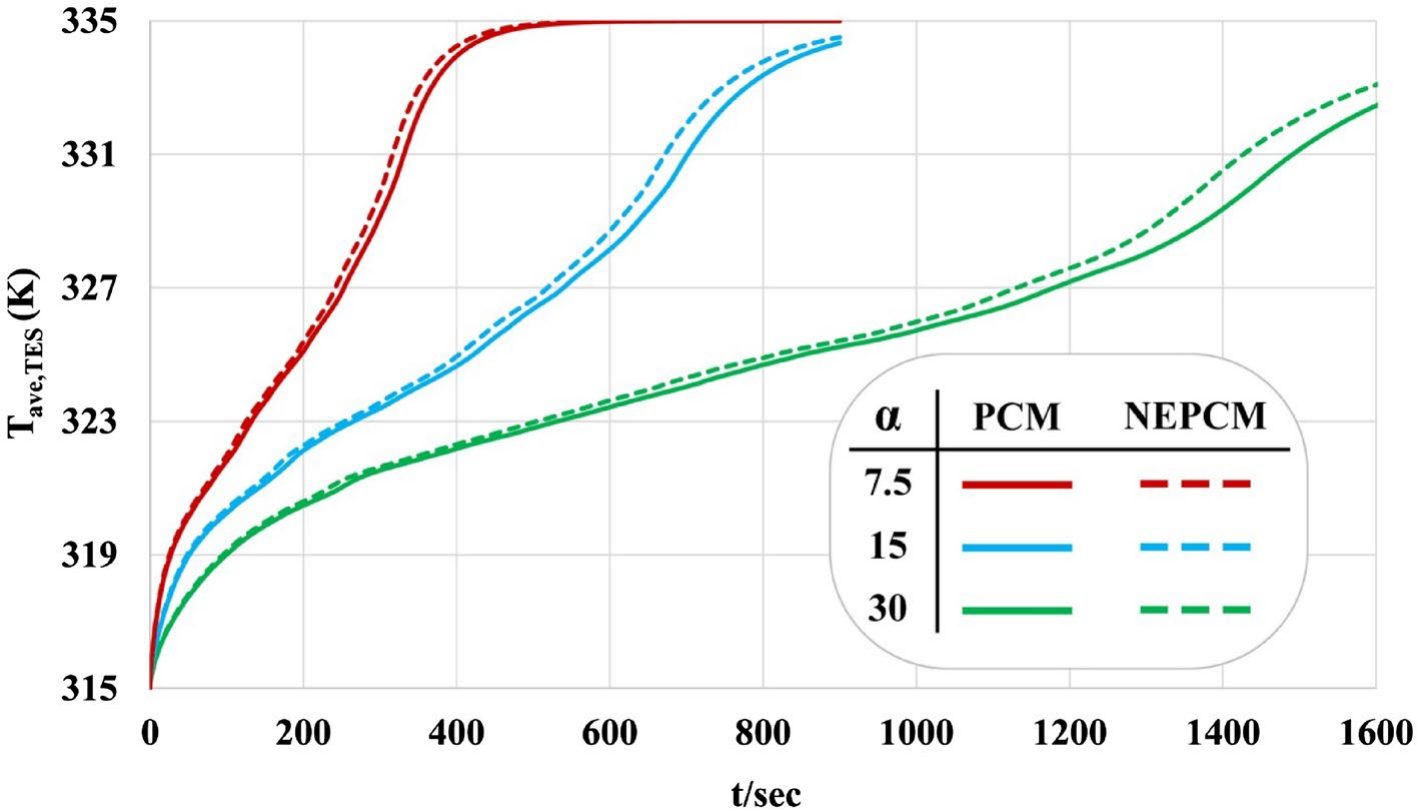
Effect of dispersing nanoparticle for the various structures in view of T_TES_.

**Figure 12:**
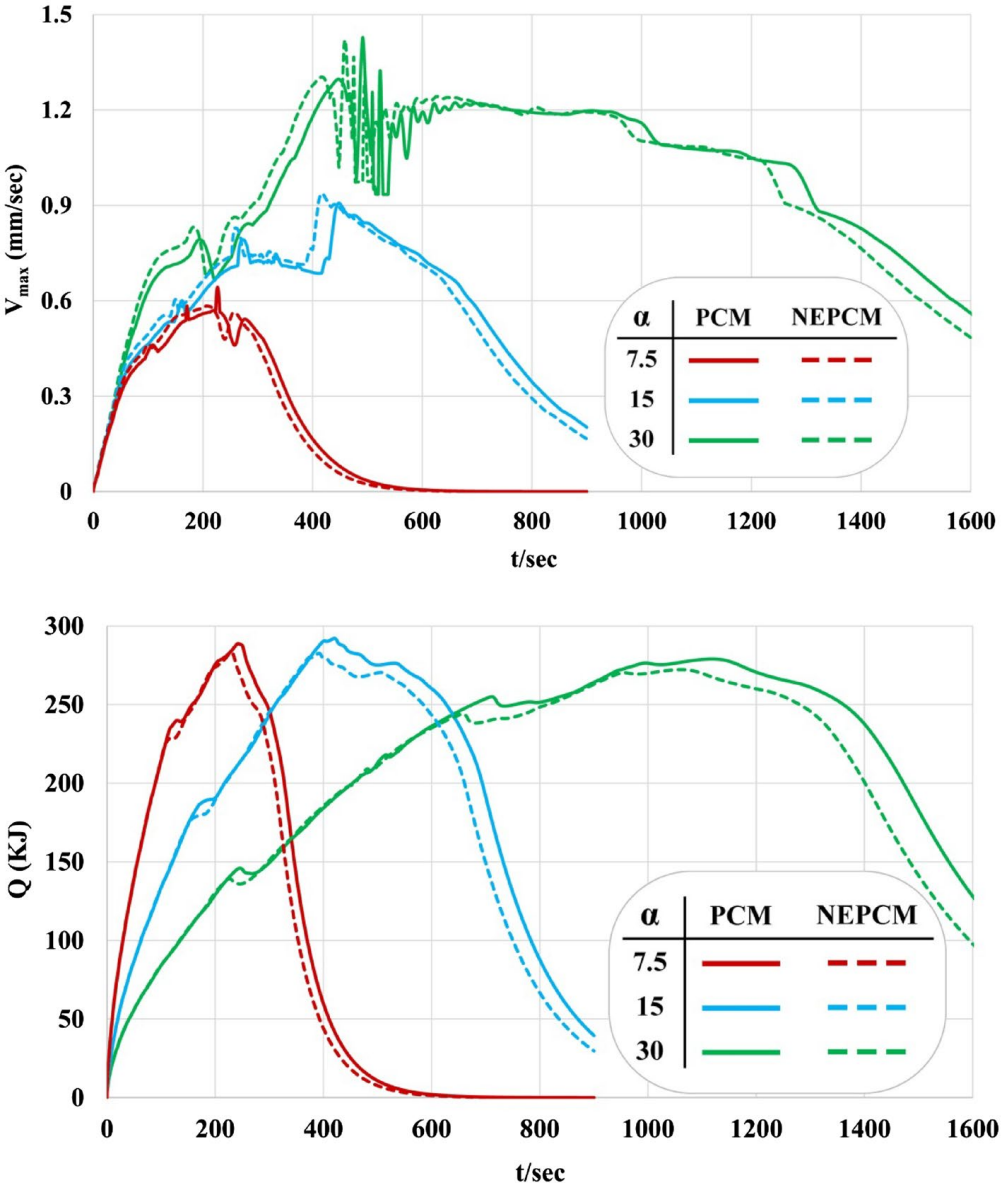
Effect of dispersing nanoparticle for the various structures in view of speed of liquid phase and Q.

In this section impacts of loading nanoparticles and changing geometry on LF, T and Q have been analyzed by reporting time-dependent profiles as demonstrated in Figs. 10, 11 and 12. Both LF and T have an increasing tendency with the rise of time. The slope of the profile of LF for α = 7.5° is much bigger than that of other geometries. Loading nano-powders can increase the rate of melting and increase the amount of LF. Loading nanomaterial leads to reduce of time about 4.73%, 4.62% and 4.98% when α = 7.5°, 15° and 30°. The influence of ϕ for α = 7.5^o^ has maximum percentage while minimum effect was reported for α = 15°. As α changes from 30° to 15° and 7.5°, the needed time reduces around 52.06% and 76.47%. The temperature of the domain reaches 335 K after the whole PCM converts to liquid because there is no heat loss. The red line in plot shows the best case in which quickest melting occurs. The maximum amount of velocity has been reported and the profile has one maximum point for each case. Due to presence of buoyancy force, the liquid paraffin can move and its velocity at first increases and then decreases. The magnitude of velocity for α = 30° is greater than that of α = 7.5°. Decreasing angle of fin makes Q increase around 149.53% and 119.18% when = 1 min and 3 min but it decreases about 48.42% when t = 6 min. Dispersing additives causes Q to increase about 150.82% and 120.08% when t = 1 min and 3 min but it declines about 47.82% when t = 6 min. With rise of time, the amount of Q firstly increases and it decreases and the time of maximum value increases with rise of angle. Loading nanomaterial makes the maximum value of Q to decrease. At the end of process, the magnitude of Q reaches zero.

## Conclusions

In present study, new design for paraffin zone has been introduced considering triplex tube. Three configuration of container have been involved and volume of paraffin is constant in all cases. To compensate the drawback of pure paraffin in absorbing heat, graphene nano-powders were loaded within the paraffin. To produce different geometries for container, angle of fin has been changed (α = 7.5°, 15° and 30°). The concentration of graphene is 0.03 which is logical range to consider single phase technique for deriving properties of NEPCM. The two dimensional domain has been considered and source terms of melting and buoyancy have been incorporated. Finite volume technique involving implicit method has been selected for simulation. The numerical procedure was verified with comparing results with previous work and low deviation of data depicts the good accommodation. Various sizes of grids have been utilized and different levels of time step to decrease the required computing price. The outputs for critical case (α = 7.5°) showed that time step should be 0.1 s and number of elements should be 294,052 which results in maximum aspect ratio of 19.8425. When t = 1 min, LF increases about 154.17% with increasing ϕ and it augments about 144.63% with decrease of α. Also, the amount of Q enhances about 150.82% and 149.53% with adding nanoparticles and utilizing lower angle, respectively. When t = 6 min, LF enhances around 109.16% with loading nanoparticles and it grows about 102.88% with decrease of angle. Besides, the magnitude of Q decreases about 47.82% and 48.42% with adding nanoparticles and utilizing lower angle, respectively. The period of melting changes from 1377 to 660 s when the angle alters from 30° to 15°. The shortest process belongs to case with α = 7.5° which takes 324 s to reach full melting. Temperature of paraffin increases with rise of time and higher volume of liquid paraffin have been achieved. The amount of Q firstly enhances and then reduces up to zero value. As α decreases from 30° to 15° and 7.5°, the completion time reduces about 52.06% and 76.47%. Dispersing nanomaterial makes period to decrease around 4.73%, 4.62% and 4.98% when α = 7.5°, 15° and 30°. The minimum impact of ϕ happens when α = 15°.

## Data Availability

All data generated or analysed during this study are included in this published article.
